# Computer-assisted evaluation of small airway disease in CT scans of Iran-Iraq war victims of chemical warfare by a locally developed software: comparison between different quantitative methods

**DOI:** 10.1186/s12880-023-01114-2

**Published:** 2023-10-23

**Authors:** Mohammad Mehdi Baradaran Mahdavi, Mehravar Rafati, Mostafa Ghanei, Masoud Arabfard

**Affiliations:** 1 Chemical Injuries Research Center, Systems Biology and Poisonings Institute, Baqiyatallah University of Medical Sciences, Tehran, Iran; 2 Department of Medical Physics and Radiology, Faculty of Paramedicine, Kashan University of Medical Sciences, Kashan, Iran

**Keywords:** Air trapping, Chemical weapons, Computed tomography, Quantitative methods, Small airway disease

## Abstract

**Objective:**

Diagnosis of small airway disease on computed tomography (CT) scans is challenging in patients with a history of chemical warfare exposure. We developed a software package based on different methodologies to identify and quantify small airway disease in CT images. The primary aim was to identify the best automatic methodology for detecting small airway disease in CT scans of Iran-Iraq War victims of chemical warfare.

**Methods:**

This retrospective case–control study enrolled 46 patients with a history of chemical warfare exposure and 27 controls with inspiratory/expiratory (I/E) CT scans and spirometry tests. Image data were automatically segmented, and inspiratory images were registered into the expiratory images' frame using the locally developed software. Parametric response mapping (PRM) and air trapping index (ATI) mapping were performed on the CT images. Conventional QCT methods, including expiratory/inspiratory mean lung attenuation (E/I MLA) ratio, normal density E/I (ND E/I) MLA ratio, attenuation volume Index (AVI), %low attenuation areas (LAA) < -856 in exhale scans, and %LAA < -950 in inhale scans were also computed. QCT measurements were correlated with spirometry results and compared across the two study groups.

**Results:**

The correlation analysis showed a significant negative relationship between three air trapping (AT) measurements (PRM, ATI, and %LAA_Exp_ < -856) and spirometry parameters (Fev1, Fvc, Fev1/Fvc, and MMEF). Moreover, %LAA_Exp_ < -856 had the highest significant negative correlation with Fev1/Fvc (*r* = -0.643, *P*-value < 0.001). Three AT measurements demonstrated a significant difference between the study groups. The E/I ratio was also significantly different between the two groups (*P*-value < 0.001). Binary logistic regression models showed PRM^Fsad^, %LAA_Exp_ < -856, and ATI as significant and strong predictors of the study outcome. Optimal cut-points for PRM^Fsad^ = 19%, %LAA_Exp_ < -856 = 23%, and ATI = 27% were identified to classify the participants into two groups with high accuracy.

**Conclusion:**

QCT methods, including PRM, ATI, and %LAA_Exp_ < -856 can greatly advance the identification and quantification of SAD in chemical warfare victims. The results should be verified in well-designed prospective studies involving a large population.

## Introduction

It is well-documented that the Iraqi government used chemical warfare against Iranians in the 1980–1988 war [1]. Sulfur mustard gas led to significant mortality among civilians in the West of Iran, and numerous veterans have struggled with its short- and long-term adverse effects, even 30 years after the incident [2]. As soon as facing sulfur mustard, victims mostly lose their vision [3], but short- and long-term adverse impacts may also occur in the skin, eyes, and respiratory system. Adverse respiratory effects are the major challenge of chemical warfare among injured veterans [4]. Chemical warfare survivors experience late pulmonary sequelae, including chronic bronchitis, bronchiolitis obliterans, pulmonary fibrosis, emphysema, and bronchiectasis [5]. Small airway disease (SAD) is the most common presentation of pulmonary injury among chemical warfare survivors [6].

Detection of early physiological impairment in the small airways is challenging. The obstruction of about 75% of all small airways is required before the appearance of changes in routine spirometry tests [7, 8]. Computed tomography (CT) provided an opportunity for early evaluation of subclinical pathological changes in the airways and lung parenchyma. SAD evaluation on CT is challenging as the airways cannot be directly visualized. Expiratory CT air trapping (AT) is regarded as specific for SAD. The AT regions are visible on expiratory phase CT as well-defined low attenuated geographic regions with contours that follow the frameworks of the affected secondary pulmonary lobules [9–11]. Visual assessment of AT is subject to the limitations inherent to intra-rater subjectivity and reliability. Furthermore, although AT could sometimes be seen as mosaic attenuation on expiration CT, diffuse AT is hard to be detected visually [12]. Meanwhile, early detection of SAD is essential to preventing irreversible damage to small airways.

Rapid advances in CT technology and the overwhelming information gained by CT have encouraged the development of CT imaging analysis software to streamline quantitative CT (QCT) into daily clinical practice for the early detection of SAD. Several quantitative methods have been introduced to detect and quantify AT on CT. Commonly used QCT methods for SAD diagnosis have traditionally used tissue volumetric summary statistics, such as the mean lung density. More advanced methods, however, classify lung fields based on a voxel-by-voxel lung attenuation change from co-registered inspiratory and expiratory CT images. Voxel-based approaches provide detailed information unattainable by more conventional QCT methods. In other words, conventional QCT methods lack the benefit of spatial localization and, therefore, cannot measure regional heterogeneity. Numerous studies have evaluated the ability of different QCT methods to identify functional SAD in chronic obstructive pulmonary disease (COPD). The ability of QCT methods to identify SAD has been demonstrated in previous studies on COPD patients [11]. However, to the best of our knowledge, no study has evaluated the application of QCT methods in patients with a history of chemical warfare exposure. Thus, this study aimed to introduce a locally developed software package including different QCT methods to evaluate SAD on CT scans of chemical warfare victims and compare different methodologies to identify the best QCT method.

## Materials and methods

### Population

This retrospective cross-sectional study was conducted on veterans exposed to chemical warfare admitted to Baqiyatallah Hospital affiliated with Baqiyatallah University of Medical Sciences (Iran) from May 2018 to February 2021.

The study protocol was first presented to the Baqiyatallah University of Medical Sciences Ethics Committee and approved (IR.BMSU.REC.1399.469). Then, the participants were briefed and reassured about the confidentiality of their personal information. They signed a written consent for participation form. All the patients underwent inspiration and expiration CT scans and spirometry. Spirometry was performed within a three-month interval from the CT scan according to the American Thoracic Society guidelines. Other clinical variables, including age, smoking status, and body mass index (BMI) were also assessed. All CT examinations were evaluated by an experienced radiologist in chest radiology for adequate inspiration and expiration, absence of significant motion artifact, and inclusion of all parts of the lung. Examinations with suboptimal quality and those with unavailable inspiration and expiration CT images were excluded. Furthermore, CT scans with any feature of acute pulmonary infection, pulmonary fibrosis, pulmonary mass, tracheobronchomalacia, or evidence of stenotic processes in the tracheobronchial tree were excluded. The other exclusion criteria were a history of lung surgery and defects in medical records. The patients were included via convenience sampling until achieving the desired sample size. Finally, 46 patients with available inspiration and expiration CT images and spirometry data were included.

Age-matched, healthy participants without a history of chemical warfare injury were also included as negative controls (*n* = 27). These participants did not smoke and had normal appearing I/E CT scans.

### Imaging technique

All the scans were obtained with an axial GE HiSpeed Advantage CT Scanner (FXI-plus; GE Medical Systems, Milwaukee, WI)**.** The scanning parameters in paired I/E CT examinations were as follows: tube voltage 120 kV; tube current 100–200 mA; field of view 350 mm; collimation 1 mm; pitch 1–1.5. The CT data were reconstructed at 1-mm slice thicknesses and 1-mm increments using a B60f kernel. The scans were obtained in the supine position, and all the participants were instructed only to take in and hold their breath in an inspiratory state. They were also instructed to exhale completely for 6–8 s and then to stop breathing at the expiratory state. No pulmonary-function-test-like coaching was used in this study. All the CT scanners were calibrated once every three months using an American Association of Physicists in Medicine standard phantom.

### Image processing

Image processing consisted of lung parenchyma segmentation, followed by deformable volumetric registration, to align the inspiration scan to the expiration scan such that the scans shared the same spatial geometry. The lungs from both CTs were segmented from the surrounding structures using a trained deep-learning U-net model described elsewhere [13]. Python 3.9 was used for developing the program.

After the generation of the segmentation maps, the inspiratory CT scans were registered to the inspiratory CT scans for each participant. The registration process was performed using the SimpleITK library based on Elastix 4.8, an open-source deformable image registration library. This algorithm iteratively optimizes the solution using mutual information with a bending energy penalty as the objective function. The optimized transformation matrix aligned the original expiratory CT scan and corresponding segmentation maps to the inspiratory geometric frame using nearest-neighbor interpolation. The 3D affine registration method was employed to register the inhale and exhale CT images of the lung based on the lung segmentation conducted in the previous step as the image mask. More details on the 3D affine registration can be found elsewhere [14].

### Conventional QCT measurements

Mean lung attenuation (MLA) was obtained by averaging the attenuation values of the voxels in the lung parenchyma. The attenuation-volume index (AVI) was calculated according to the following expression: (MLAin expiration − MLA in inspiration)/volume decrease ratio (VDR). The VDR was calculated using the following expression: 100 × (lung volume in inspiration − lung volume in expiration)/lung volume in inspiration. Besides, the E/I ratio and ND E/I ratio were used as conventional measurements. The E/I ratio was defined as the ratio of MLA at expiration/inspiration. The ND E/I ratio was defined as the ratio of MLA at expiration/inspiration in seemingly normal lung regions (voxels greater than − 950 HU at end-inspiration and greater than − 856 HU at end-expiration). The percentages of voxels with values lower than -856 HU on the expiratory scan and less than -950 HU on the inspiration scan were obtained to examine the extent of AT and emphysema, respectively.

### ATI mapping

In ATI mapping, the lung parenchyma was classified into two lung regions: 1) regions with AT, and 2) emphysematous regions (Emph). Emph was defined as the volume fraction of voxels with a radio density of less than − 950 HU on inspiration CT. AT was presented as the volume fraction of voxels with a change in radio-density of less than 60 HU between inspiration and co-registered expiration CT scans in the lung parenchyma sufficiently inflated on inspiration CT with a lung density of -856 to -950 HU. The AT region was presented as its volume relative to the total included inspiratory parenchyma with a radio-density of -856 to -950 HU.

### Parametric Response Map (PRM)

Following lung segmentation and image registration, paired histograms of both inspiratory and expiratory CT scans were analyzed. PRM classified each voxel based on its attenuation values considering the traditional fixed thresholds of -950 HU for emphysema in the inspiratory CT and -856 HU in the expiratory CT for defining AT. Any voxel with inspiratory CT < -950 HU and expiratory CT < -856 represented emphysema (PRM^Emph^); all voxels > -950 HU in the inspiratory CT and < -856 HU in the expiratory CT represented AT as functional regions of SAD (PRM^Fsad^); and all voxels above both thresholds in both scans represented normal lung regions (PRM^Norml^).

**Fig. 1:**
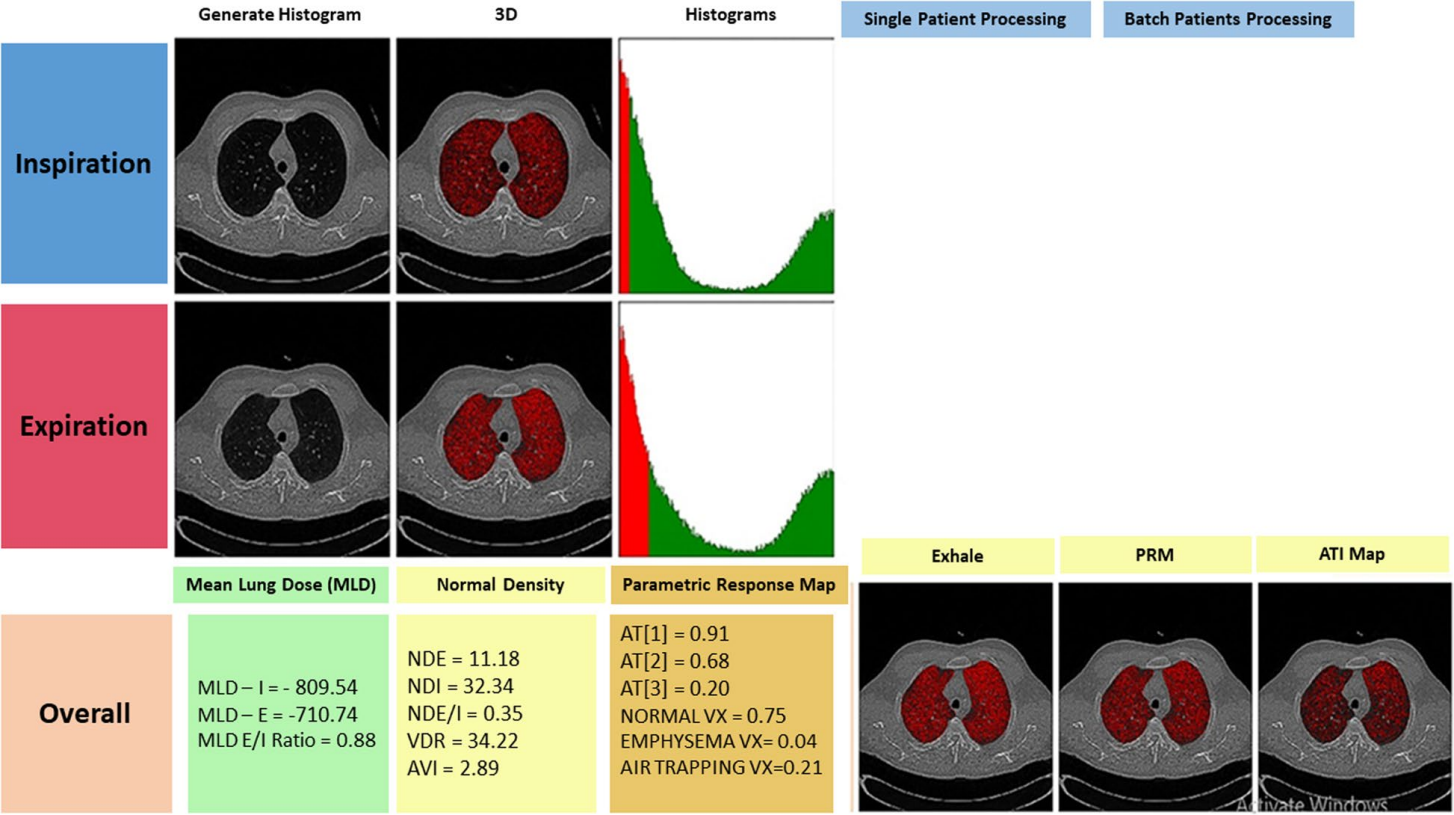
Example of a CT scan of a patient interpreted by the BLADQA

Figure 1 presents the output of the locally developed software, Baqiyatallah Lung Analyzer (BLADQA), to detect and quantify air trapping.

### Statistical analysis

The data were analyzed by IBM SPSS 26. Pearson correlation analysis was used to assess the association between QCT measurements and spirometry parameters. The correlations were compared using Fisher's r-to-z transformation [15]. Scatter plots and correlation analysis were applied to evaluate the relationship between emphysema and AT measurement classes (PRM^Fsad^ -ATI-%LAA_Exp_ < -856 HU). An independent samples t-test was applied to compare the QCT measurements between the two groups. A binary logistic regression model and receiver operating characteristic (ROC) analysis were used to correlate the AT measurements with the likelihood that an individual would be in the patient group. The Bonferroni correction method was applied for adjustment in multiple testing. The significance level was < 0.05.

## Results

In this study, 73 men participated, including 46 (63.0%) in the patient group and 27 (37.0%) in the control group. The participants' baseline characteristics are described in Table 1.

**Table 1:** Baseline participant’s characteristics

Variables	Groups of Study	
	Case *n* = 46	Control *n* = 27
Age (years)	53.78 ± 9.43	52.33 ± 12.55
BMI (Kg/m^2^)	26.57 ± 4.00	26.24 ± 2.27
Fvc (%)	72.73 ± 20.78	92.11 ± 15.90
Fev1 (%)	79.84 ± 24.23	91.42 ± 14.89
Fev1/Fvc (%)	79.81 ± 10.93	88.56 ± 11.97
MMEF (%)	82.90 ± 37.31	89.87 ± 12.50
Smoking (Yes/No)^*^	8(17.40%)	0(0%)

*Fev1/Fvc* Ratio of forced expiratory volume in 1 s to forced vital capacity, *MMEF* Maximal mid-expiratory flow

^*^n (%) for categorical variable

### Association of QCT measurements with spirometry results

Table 2 shows the association of all QCT measurements with spirometry parameters. There was a significant negative correlation between PRM^Fsad^ and Fev1/Fvc (*r* = -0.610, *P*-value < 0.001). Moreover, a significant negative correlation was found between ATI and Fev1/Fvc (*r* = -0.602, *P*-value < 0.001). The best correlation was observed between %LAA_Exp_ < -856 and Fev1/Fvc (*r* = -0.665, *P*-value < 0.001). Besides, PRM^Fsad^ + PRM^Emph^ demonstrated a strong negative correlation with Fev1/Fvc (*r* = -0.617, *P*-value < 0.001). The E/I ratio had a moderate negative correlation with Fev1/Fvc and MEEF (*r* = -0.429 and *r* = -0.323, respectively, with *P*-value < 0.01). The results revealed a significant negative correlation between PRM^Emph^ and Fev1/Fvc (*r* = -0.445, *P*-value < 0.001). In addition, there was a significant negative correlation between %LAA_ins_ < -950 and Fev1/Fvc (*r* = -0.506, *P*-value < 0.001; Table 2).

**Table 2:** Pearson correlation coefficient between QCT measurements and Spirometry parameters for both groups of study

Variables r(*P*-value)^$^	Fvc	Fev1	Fev1/Fvc	MMEF
PRM^Fsad^	-0.400(< 0.001)^b^	-0.422(< 0.001)^b^	-0.610(< 0.001)^b^	-0.388(0.001)^b^
ATI	-0.395(0.001)^b^	-0.438(< 0.001)^b^	-0.602(0 < 001)^b^	-0.351(0.002)^b^
%LAA_Exp_ < -856	-0.437(< 0.001) ^b^	-0.508(< 0.001)^b^	-0.665(< 0.001)^b^	-0.432(< 0.001)^b^
PRM^Fsad^ + PRM^Emph^	-0.446(< 0.001) ^b^	-0.527(< 0.001)^b^	-0.617(< 0.001)^b^	-0.452(< 0.001)^b^
E/I ratio	-0.438(< 0.001)^b^	-0.397(0.001)^b^	-0.459(< 0.001)^b^	-0.323(0.005)^b^
ND E/I ratio	0.308(0.014)^a^	0.290(0.021)^a^	0.122(0.340)	0.275(0.029)^a^
AV/I ratio	0.100(0.412)	0.100(0.411)	0.348(0.003) ^b^	0.081(0.507)
PRM^Emph^	-0.353(0.002)^b^	-0.457(< 0.001)^b^	-0.445(< 0.001)^b^	-0.373(0.001)^b^
%LAA_ins_ < -950	-0.133(0.270)	-0.204(0.088)	-0.506 (< 0.001)^b^	-0.213(0.075)

^$^*P*-value calculated from Pearson Correlation Coefficient Analysis

^a^correlation is significant at the 0.05 level

^b^correlation is significant at the 0.01 level

Univariate linear regression analysis confirmed %LAA_Exp_ < -856 as a significant predictor of Fev1/Fvc (β _%LAAExp<-856_ = -96.02, *P*-value = 0.002). Also, univariate linear regression analysis confirmed that ATI and %LAA_Exp_ < -856 were significant predictors of MMEF (β_ATI_ = 224.73, *P*-value = 0.033, β _%LAAExp<-856_ = -253.23, *P*-value = 0.007, respectively).

### Association of air trapping indices with emphysema

**Fig. 2:**
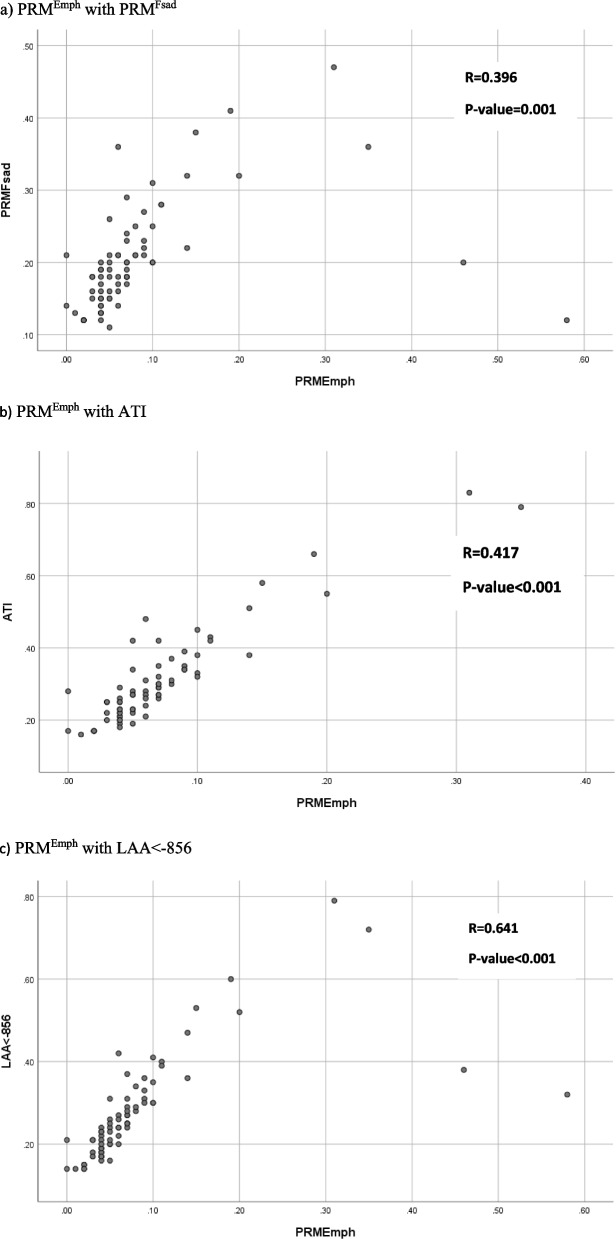
Scatter plot of PRM^Emph^ with three classes of air trapping (PRM^Fsad^ -ATI-LAA < -856)

To illustrate the association between PRM^Emph^ and air trapping, a scatter plot and linear correlation coefficient were used. All the graphs showed a straight line with a positive slope. There was a positive and moderate relationship between PRM^Emph^ and ATI (*r* = 0.417, *P*-value < 0.001). Significant relationships were also observed between PRM^Emph^ and %LAA_Exp_ < -856 and between PRM^Emph^ and PRM^Fsad^ (*r* = 0.641, *P*-value < 0.001, *r* = 0.396, *P*-value = 0.001, respectively). All the mentioned correlations in any pair were statistically different from each other (*P*-value < 0.001; Fig. 2).

**Fig. 3:**
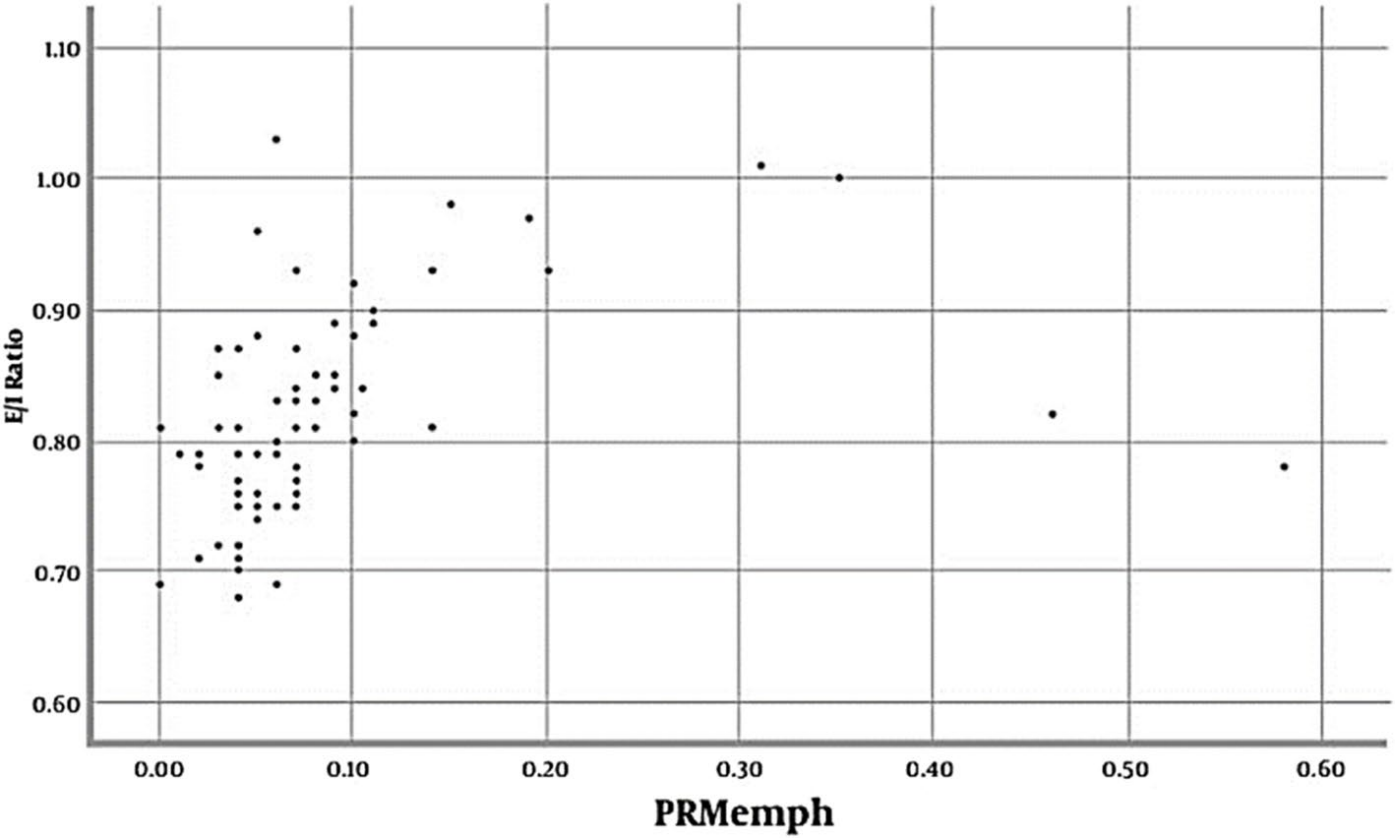
Scatter plot of PRM^Emph^ with the E/I ratio

The scatter plot in Fig. 3 displays the significant positive relationship between PRM^Emph^ and E/I ratio (*r* = 0.373, *P*-value = 0.001).

Similarly, the Pearson correlation test was applied to assess the association between %LAA_ins_ < -950, as a measure of emphysema, and three classes of air trapping (PRM^Fsad^, ATI, and %LAA_Exp_ < -856). The correlation between %LAA_ins_ < -950 and %LAA_Exp_ < -856 was significant and positive (*r* = 0.526, *P*-value < 0.001). In addition, %LAA_ins_ < -950 significantly correlated with PRM^Fsad^ and ATI (r = 0.373, *P*-value = 0.001, *r* = 0.462, *P*-value < 0.001, respectively).

### Comparison of three classes of air trapping measurement

**Fig. 4:**
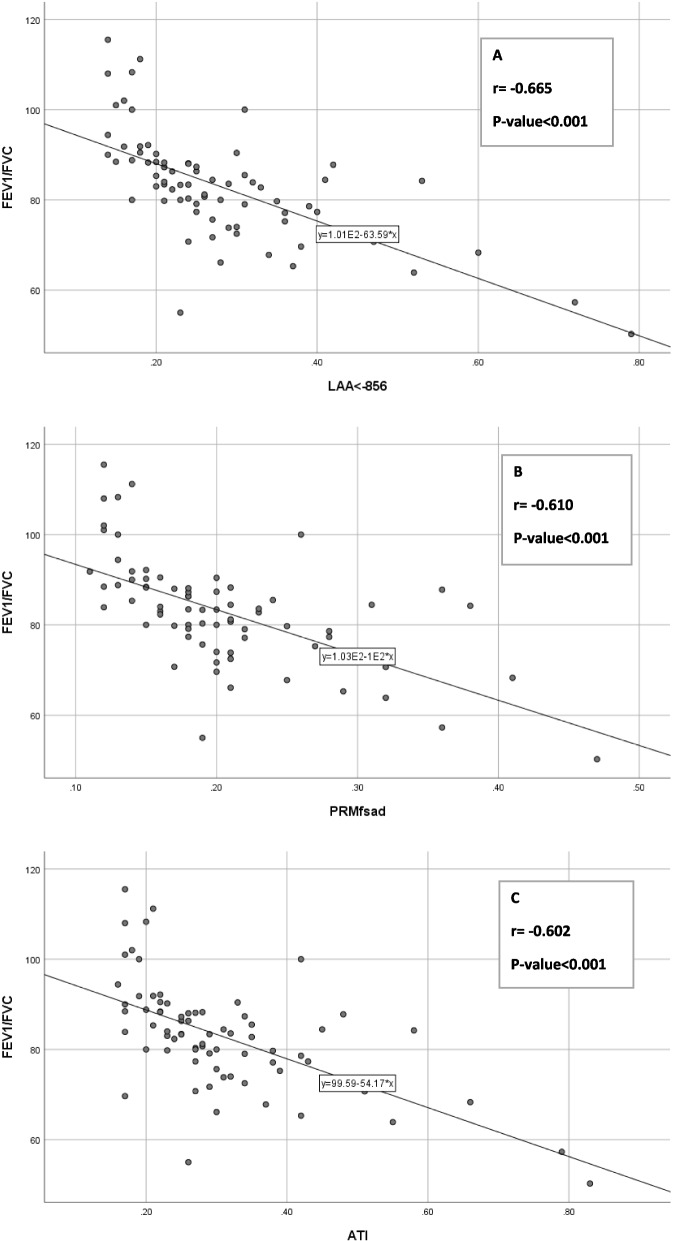
Scatter plots of three AT measurement methods with Fev1/Fvc: Correlation between %LAA_Exp_ < -856 and Fev1/Fvc (A; R^2^ = 0.442), Correlation between PRM^Fsad^ and Fev1/Fvc (B; R^2^ = 0.372), Correlation between ATI and Fev1/Fvc (C; R^2^ = 0.363)

Graphs and correlation tests were used to compare the correlation statistics with Fev1/Fvc across three classes of AT measurement (Fig. 4).

Table 2 presents the significant negative correlation between the three classes of AT measurement and Fev1/Fvc. There were significant differences in correlation coefficients for the pairs of %LAA_Exp_ < -856-Fev1/Fvc and PRM^Fsad^-Fev1/Fvc (*r* = -0.665 and *r* = -0.610, respectively; *P*-value = 0.043) and for the pairs of %LAA_Exp_ < -856-Fev1/Fvc and ATI-Fev1/Fvc (*r* = -0.665, *r* = -0.602, respectively; *P*-value = 0.010). However, there was no significant difference in the correlation coefficient for the pairs of PRM^Fsad^-Fev1/Fvc and ATI-Fev1/Fvc (*r* = -0.544, *r* = -0.606, respectively; *P*-value = 0.385).

Table 2 indicates the significant negative correlation between three classes of AT measurement and MMEF, but there were no significant differences in correlation coefficients for the pairs of %LAA_Exp_ < -856-MMEF and PRM^Fsad^-MMEF (*r* = -0.432, *r* = -0.388, respectively; *P*-value = 0.125) and the pairs of PRM^Fsad^-MMEF and ATI-MMEF (*r* = -0.388, *r* = -0.351, respectively; *P*-value = 0.125). However, there was a significant difference in the correlation coefficient for the pairs of %LAA_Exp_ < -856-MMEF and ATI-MMEF (*r* = -0.432, ***r*** = -0.351, respectively; *P*-value = 0.006).

### Comparison of QCT measurements between the two groups

Table 3 compares QCT measurements between the two groups. The t-test results showed that for conventional methods, only the E/I ratio was statistically different between the two groups (*P*-value < 0.001). Moreover, PRM^Emph^ significantly differed between the patient and control groups (*P*-value < 0.001). All the AT measurements (PRM^Fsad^, %LAA_Exp_ < -856, and ATI) significantly differed between the case and control groups (*P*-value < 0.001; Table 3).

**Table 3:** Comparisons of QCT measurements between case and control groups

Variables	Case	Control	95% CI of mean difference^a^		*P*-value^*^
	Mean ± SD	Mean ± SD			
E/I ratio	0.85 ± 0.08	0.77 ± 0.05	0.05	0.11	< 0.001
ND E/I ratio	0.86 ± 0.56	1.23 ± 1.88	-1.20	0.45	0.365
AVI ratio	3.38 ± 1.33	3.87 ± 0.83	-1.06	0.08	0.092
PRM^Emph^	0.10 ± 0.11	0.05 ± 0.02	0.02	0.09	0.002
%LAA_ins_ < -950	0.29 ± 0.08	0.27 ± 0.09	-0.01	0.06	0.152
PRM^Fsad^	0.23 ± 0.08	0.16 ± 0.03	0.04	0.09	< 0.001
%LAA_Exp_ < -856	0.32 ± 0.13	0.21 ± 0.05	0.06	0.15	< 0.001
ATI	0.35 ± 0.15	0.24 ± 0.05	0.06	0.16	< 0.001

^*^P-value calculated from two independent sample t-test

^a^95% Confidence Interval

**Fig. 5:**
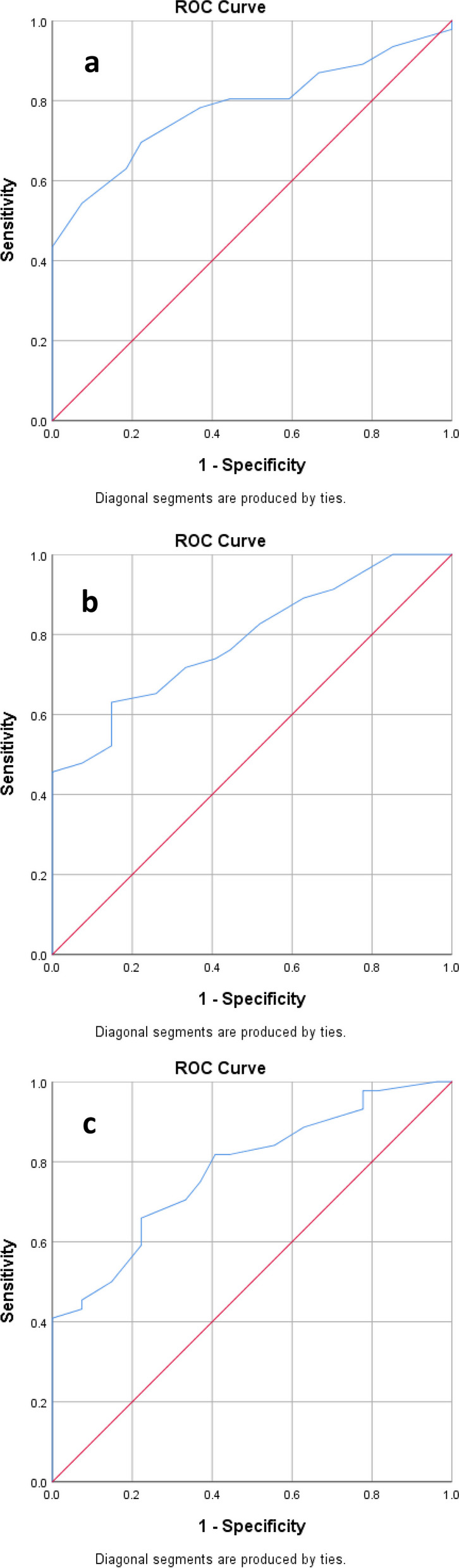
**a** ROC curve to illustrate diagnostic ability of logistic regression model, based on PRM^Fsad^ as a predictor. **b** ROC curve to illustrate the diagnostic ability of the logistic regression model, based on %LAA_Exp_ < -856 as a predictor. **c** ROC curve to illustrate diagnostic ability of logistic regression model, based on ATI as a predictor

A binary logistic regression model was applied to correlate PRM^Fsad^ with the likelihood of a participant being in the patient group. Age and sex were matched in both groups because all the participants were male, and the t-test showed no significant difference in age between the patient and control groups (*P*-value = 0.577). The logistic regression model demonstrated the significant effect of PRM^Fsad^ after adjusting for emphysema as a confounder (OR_adj_ = 1.30, *P*-value = 0.001), so PRM^Fsad^ was approved as a significant predictor of the outcome (being a patient). Then, to find an optimal cut-point to classify the participants into the case and control groups, the ROC curve analysis was applied using PRM^Fsad^ as an independent variable (Fig. 5a and Table 4). PRM^Fsad^ significantly identified patients with an area under the ROC curve of 0.80 (*P*-value < 0.001; Table 4). ROC analysis generated an optimal PRM^Fsad^ cut-point of 19% of the total lung volume. Values equal to or greater than 19% of PRM^Fsad^ identified patients with a sensitivity of 0.78 and specificity of 0.70.

**Table 4:** Area under ROC curve

Area	Standard Error	95% CI for Area		*P*-value
0.80	0.05	0.69	0.89	< 0.001
0.79	0.05	0.68	0.88	< 0.001
0.78	0.05	0.67	0.88	< 0.001

The binary logistic regression model also showed the significant impact of %LAA_Exp_ < -856 on the outcome, adjusted for emphysema as a confounder (OR_adj_ = 1.18, *P*-value = 0.001). Next, ROC analysis using %LAA_Exp_ < -856 as a strong predictor was performed to find an optimal cut-point for classifying the participants into the two groups (Fig. 5b).

Table 4 (The second row) shows the significant and valuable accuracy of the logistic model based on %LAA_Exp_ < -856 as a significant predictor (AUC = 0.79, *P*-value < 0.001). ROC analysis identified the value of 0.23 as an %LAA_Exp_ < -856 optimal cut-point with a sensitivity of 0.72 and specificity of 0.70. The %LAA_Exp_ < -856 value > 0.23 of the total lung volume assigned the participants to the patient group.

Similarly, the binary logistic regression model expressed the significant impact of ATI on the outcome, adjusted for emphysema as a confounder (OR_adj_ = 1.16, *P*-value = 0.001). Subsequently, ROC analysis using ATI as a relatively strong predictor was performed to find an optimal cut-point to classify the participants into two groups (Fig. 5c).

Table 4 (The third row) lists the significant and valuable accuracy of the logistic model based on ATI as a significant predictor (AUC = 0.78, *P*-value < 0.001). ROC analysis identified the value of 0.27 as an ATI optimal cut-point with a sensitivity of 0.70 and specificity of 0.70. The ATI value > 0.27 of lung volume with radio-density of -856 to -950 HU in inhaled CT assigned the participants into the patient group.

**Fig. 6:**
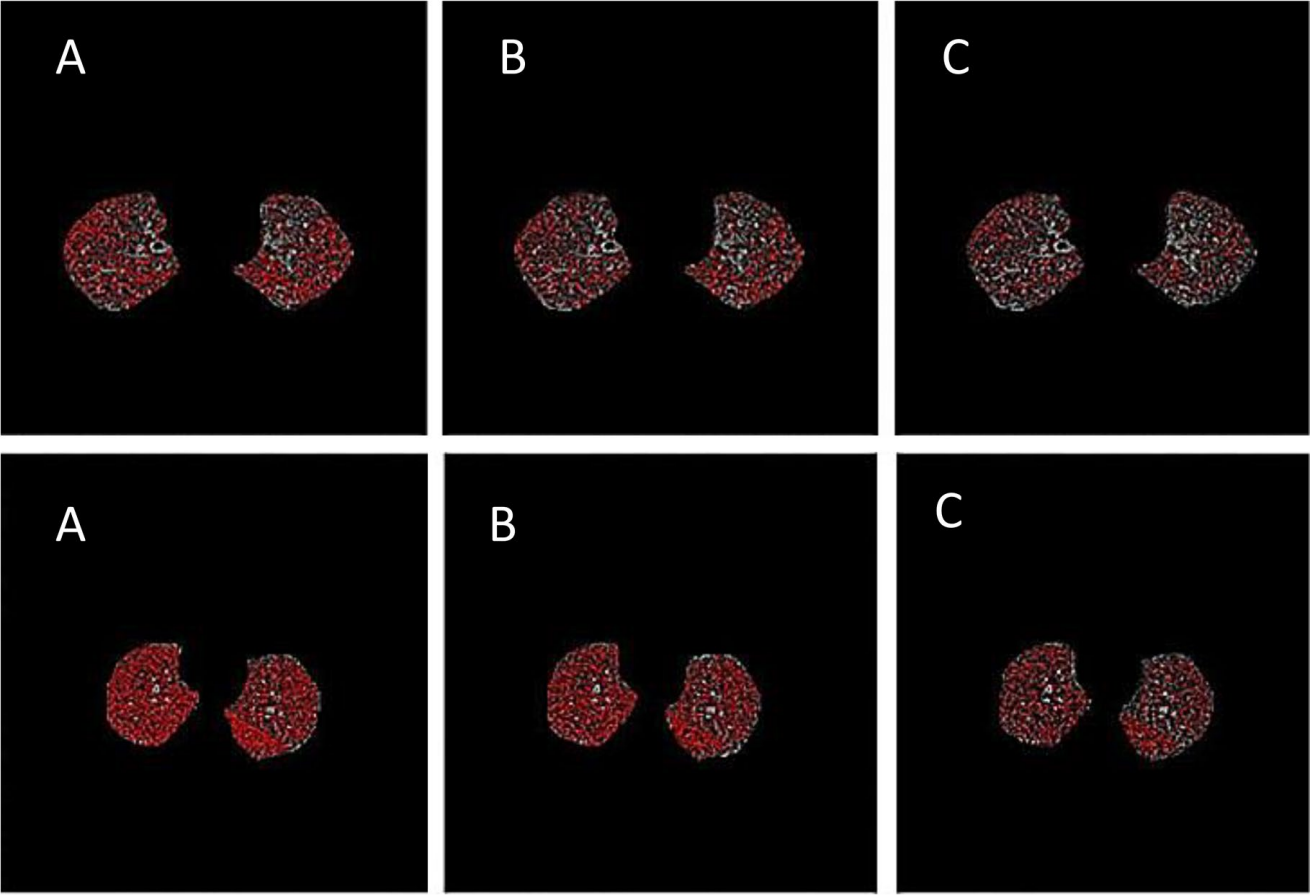
AT maps from A) static threshold of -856HU, B) the Parametric Response Map(PRM), and C) the Air Trapping Index(ATI) method in a patient (lower row), and control subject (upper row)

The ability of PRM^Fsad^, ATI, and %LAA_Exp_ < -856 to characterize AT is demonstrated in representative CT images in a patient and a control participant (Fig. 6).

## Discussion

During clinical investigations of chemical warfare victims of the Iraq-Iran War, a high proportion of symptomatic and asymptomatic participants was discovered with features of air trapping on chest HRCT [5]. As known, traditional visual assessment of AT is inherent to unreliability and intra-rater subjectivity, particularly when follow-up CTs are needed to evaluate the efficacy of therapeutic interventions [16]. On the other hand, accurate visual identification and quantification of AT requires a trained radiologist to contour AT areas, a time-consuming and arduous approach [17]. The optimal QCT method for identifying and quantifying AT is still under debate. The present study aimed to evaluate different quantitative methods in identifying and quantifying AT and compare the values between the patients and healthy participants. To the best of our knowledge, this is the first study using computer-aided methods to evaluate the late pulmonary complications of chemical warfare victims.

Small airways cannot be directly visualized using current radiologic techniques. However, it has been revealed that pulmonary densitometry parameters measured based on expiratory CT scans allow the indirect evaluation of airflow limitation. These quantitative methods are based on detecting AT as regions of LAA. The LAA is a measure of lung density, defined as the percentage of voxels in the lung below a predefined threshold. Fully inflated normal alveoli have an average attenuation of − 856 HU, and with a proper expiratory effort, the average attenuation increases approximately by 150 HU. Regions with attenuation of less than -856 HU on expiratory CT may indicate inadequate gas emptying [18–20]. In prior studies, it was revealed that %LAA_Exp_ < -856 significantly correlates with spirometry findings [20]. In line with the previous studies, our study reported a significant positive correlation between %LAA_Exp_ < -856 and spirometry parameters. Furthermore, our study revealed that this measurement is an excellent predictor of airflow obstruction in spirometry and could reliably discriminate between the case and control groups.

The proportion of mean lung attenuation from the density histogram on expiratory CT to the one on inspiratory scan is called the expiratory to inspiratory ratio of mean lung attenuation (E/I MLA) and is presented as a percentage. As predicted, more severe AT leads to a higher E/I MLA ratio. Multiple studies have used this parameter to evaluate small airway obstruction, and the findings express its acceptable performance [21, 22]. E/I MLD is most suitable for detecting AT among indirect QCT measurements, including %LAA_Exp_ < -856 and relative volume change_-860- -950_ [23]. The present study showed a significant moderate correlation between the E/I ratio and spirometry findings and suggests that the E/I ratio could be a reliable method in repeat CT examinations for patient follow-up.

According to the literature, in the presence of moderate to severe emphysema, no significant correlation is found between airflow limitation and changes of %LAA_Exp_ < -856 from expiratory to inspiratory CT. Meanwhile, a significant correlation is seen between airflow obstruction and changes of %LAA_Exp_ < -856 in the presence of mild forms of emphysema [18]. The patients in the present study had a mild form of emphysema; therefore, a strong correlation of %LAA_Exp_ < -856 aligns with the aforementioned principles.

Since the E/I ratio can be greatly affected by the difference in the amount of inspiration, Nagatani et al. proposed a new index called AVI that can be adjusted for respiratory level. The authors showed that AVI is less influenced by variations in VDR than other conventional indices of AT measurement. AVI is defined as the increase in MLA divided by VDR [24]. We included AVI in our software package to adjust the influence of the inspiration amount on the E/I ratio. The results revealed no difference in AVI between the case and control groups and no significant correlation between AVI and spirometry findings. The CT index of global AT is the average of regional AT, so lung regions with coexisting normal and AT regions may be missed by evaluating the entire lung. Therefore, we recommend the evaluation of AVI in each lobe separately in future studies.

Some victims of chemical weapons with normal appearing imaging or pulmonary function test results were shown to have bronchiolitis obliterans based on lung biopsy [5]. It has been proposed that areas of the lung that appear normal using conventional CT measurements may have mild disease. Sandeep et al. hypothesized that the regions with normal lung density defined by predefined thresholds (voxels greater than − 910 HU at end-inspiration and greater than − 856 HU at end-expiration) contain areas with subthreshold air trapping, and the E/I ratio in these seemingly normal regions (ND E/I) may help detect SAD that is not detectable by more conventional quantitative measurements. They showed that the ND E/I ratio is independently associated with airflow obstruction, BMI, and FEV1 changes on follow-up [25]. Our study found no relationship between the ND E/I ratio and spirometry findings and no significant difference in computed values between the patient and control groups. Nevertheless, studies that include symptomatic patients with normal I/E CT exams may better show the usefulness of this index.

A parametric response map (PRM) is a novel CT-based metric for visualizing and quantifying emphysema and SAD. In a voxel-based image analysis technique including both inspiratory and expiratory CT scans, PRM provides a global evaluation of the lung with a color map representing normal lung tissue, SAD, and emphysema [26–28]. Vasilescu et al. correlated ex vivo PRM to in vivo lung tissue measurements and concluded that PRM^Fsad^ identifies lung tissue with SAD. PRM is strongly associated with the presence and severity of COPD and can thus be a valuable imaging biomarker to classify COPD phenotypes [29]. Galban et al. evaluated PRM for detecting bronchiolitis obliterans post-hematopoietic stem cell transplantation. The results showed that the mean PRM^Fsad^ was significantly greater in patients with bronchiolitis obliterans than in age-matched control participants [30]. Our study showed consistent findings in chemical warfare victims, as PRM^Fsad^ strongly predicted being in the patient group.

Since PRM considers only slight dynamic density changes for each voxel, we added another method called ATI to our software package [31]. Following the global alignment of inhale and exhale CT images in the ATI method, the changes in attenuation values from expiratory to inspiratory CT are calculated for each voxel. When the volume of voxels has a subtraction value lower than the predefined threshold, it is determined as the volume fraction of AT. A specific threshold with the strongest correlation with spirometry findings is considered the optimal subtraction value to assess the severity of AT. Lee demonstrated 60 HU as the optimal CT threshold of the subtraction method for AT [32]. Barbosa considered the range of 25–75 HU as the optimal subtraction threshold [16]. Kim et al. considered a density difference of less than 50 HU to determine regions with AT [32]. Because density difference is impacted by the baseline inflation of the lung on inspiration, Hwang et al. recently proposed a similar method. They defined AT regions as the volume fraction of voxels exhibiting a density change of less than 60 HU between inspiration and expiration CTs only in lung voxels sufficiently inflated on inspiration CT with a radio density of lower than -856 HU. Unlike PRM, the ATI method proposed by Hwang et al. detects AT in both normal and emphysematous regions. Hwang et al. demonstrated that modified ATI may have a better function than PRM^Fsad^ to characterize SAD [31]. In the present study, we included only regions with radio density of -856 to -950 HU, assuming that emphysematous regions have no major contribution to SAD. Our study showed a good correlation between ATI and spirometry parameters. Besides, ATI was a good predictor of outcomes in our study. As known, the optimal threshold for identifying AT regions depends on the CT machines model, slice thickness, and reconstruction algorithm; thus, we recommend that future studies test different threshold values to test other subtraction values.

In our study, AT measurement indices were correlated with emphysema (both PRM^Emph^ and %LAA_insp_ < -950) to determine the degree of association with pulmonary emphysema. The extent of emphysema less affected PRM^Fsad^ compared to ATI and %LAA_Exp_ < -856 because of the weaker correlation of PRM^Fsad^ with emphysema. In addition, %LAA_Exp_ < -856 was influenced less than ATI by the extent of emphysema. Considering the aforementioned finding, PRM^Fsad^ may be a better measurement for SAD in the presence of significant emphysematous variations.

Our study was not free from limitations. This was a retrospective study with inherent shortcomings, including a small population. Another limitation is that imaging protocols were not strictly controlled. Although all the CT examinations were made in a single center with the same CT machine and the same imaging protocol, the CT parameters may be impacted by non-standardized protocols. In addition, no spirometric control was used in our study and, therefore, the quantified AT values in our study may be impacted by inappropriate inflation and deflation levels executed during the respiratory cycle. However, necessary care was taken for CT examination, and this study only included I/E CTs that had been verified for proper inspiratory/expiratory efforts by an experienced radiologist in thoracic imaging.

## Conclusion

We focused on the correlation of different QCT metrics with pulmonary function tests and the corresponding differences between the case and control groups. The study reported promising findings for %LAA_Exp_ < -856, PRM^Fsad^, and ATI. Since about 30 years had passed since the chemical weapon exposure of our cases, different kinds of equipment were used for their evaluation. Hence, prospective studies with detailed standardized CT protocols and a larger population are mandatory to confirm the results of the current study. Furthermore, the potential application of these methods for follow-up or treatment response evaluation should be assessed.

## Data Availability

The datasets used during the current study are available from the corresponding author on reasonable request.
